# Preclinical efficacy and immunogenicity assessment to show that a chimeric *Plasmodium falciparum* UB05‐09 antigen could be a malaria vaccine candidate

**DOI:** 10.1111/pim.12514

**Published:** 2018-01-25

**Authors:** J. N. Dinga, S. D. Gamua, S. M. Ghogomu, V. P. K. Titanji

**Affiliations:** ^1^ Biotechnology Unit Faculty of Science University of Buea Buea Cameroon; ^2^ Department of Biochemistry and Molecular Biology Faculty of Science University of Buea Buea Cameroon; ^3^ Faculty of Science, Engineering and Technology Cameroon Christian University Institute Bali Cameroon

**Keywords:** circumsporozoite protein‐related antigen, exported protein 1, growth inhibition assay, immunization, malaria vaccine candidate, UB05 antigen, UB05‐09

## Abstract

Although it is generally agreed that an effective vaccine would greatly accelerate the control of malaria, the lone registered malaria vaccine Mosquirix™ has an efficacy of 30%‐60% that wanes rapidly, indicating a need for improved second‐generation malaria vaccines. Previous studies suggested that immune responses to a chimeric *Plasmodium falciparum* antigen UB05‐09 are associated with immune protection against malaria. Herein, the preclinical efficacy and immunogenicity of UB05‐09 are tested. Growth inhibition assay was employed to measure the effect of anti‐UB05‐09 antibodies on *P. falciparum* growth in vitro. BALB/c mice were immunized with UB05‐09 and challenged with the lethal *Plasmodium yoelii* 17XL infection. ELISA was used to measure antigen‐specific antibody production. ELISPOT assays were employed to measure interferon‐gamma production ex vivo after stimulation with chimeric UB05‐09 and its constituent antigens. Purified immunoglobulins raised in rabbits against UB05‐09 significantly inhibited *P. falciparum* growth in vitro compared to that of its respective constituent antigens. A combination of antibodies to UB05‐09 and the apical membrane antigen (AMA1) completely inhibited *P. falciparum* growth in culture. Immunization of BALB/c mice with recombinant UB05‐09 blocked parasitaemia and protected them against lethal *P. yoelii* 17XL challenge infection. These data suggest that UB05‐09 is a malaria vaccine candidate that could be developed further and used in conjunction with AMA1 to create a potent malaria vaccine.

## 1 INTRODUCTION

Despite the considerable success of the global effort to control malaria, it still claimed 429 000 lives in 2015.^1^ Meanwhile, the main control measures like arteminisin‐based combination therapy, the use of impregnated mosquito bed nets and the targeted use of insecticides are threatened by the development and spread of drug and insecticide resistances. Conceivably, the use of an effective vaccine against malaria would accelerate the control and eventual elimination of this ancient scourge.

At least 3 different strategies to develop a malaria vaccine have been identified as follows: the pre‐erythrocytic stage targeted by a infection‐blocking vaccine, the erythrocytic stage targeted by an antimorbidity vaccine and the sexual stage targeted by a transmission‐blocking vaccine.^2^ Recently, it has been proposed that an effective vaccine against malaria would be required to target many stages of the parasite life cycle.^3^ The recently approved malaria vaccine, Mosquirix™, which was previously called RTS,S was derived from the circumsporozoite protein (CSP). It targets the initial infection of the liver and has shown partial efficacy^4^ that wanes rapidly.^5^ As the clinical symptoms associated with malaria are attributed to the asexual stages of the parasite life cycle, it is desirable that a subunit vaccine against malaria should also contain blood‐stage components.

Humans living in endemic regions have been known to acquire protection from symptomatic malaria after repeated exposure over years. Blood‐stage antigens have been shown to be targeted by antibodies, and this phenomenon is believed to have contributed at least in part to this naturally acquired immunity.^6^ This has been demonstrated by passive transfer of immunity studies, in which the transfer of total immunoglobulins from individuals led to very substantial reductions of parasitaemia and clinical symptoms thereby implicating Immunoglobulin‐G in the acquisition of protective immunity to malaria.^7^

Currently, a number of blood‐stage antigens are being included as vaccine components in clinical development.^8^ The most studied and advanced blood‐stage vaccine antigens include the merozoite surface protein 3 (MSP‐3), serine repeat antigen 5 (SERA‐5) and apical membrane antigen 1 (AMA1).^8^ These candidates have not been efficacious in African children.^9^, ^10^ However, a multistage vaccine made up of AMA1 and CSP components reduced the incidence of clinical malaria episodes in vaccinated children by 50% compared to those in the control group.^11^ The extensive genetic polymorphisms seen in the current malaria vaccine candidates are considered to be a major obstacle for blood‐stage vaccine development.^12^ Even though a number of blood‐stage antigens are under vaccine development, it is still possible and necessary to search for more subunit vaccine candidates to improve the outcome of vaccination. While reverse vaccinology^13^, ^14^ and the use of orthologous proteins^15^, ^16^ are methods that have been used to identify antigens that are involved in host‐parasite interactions, it would be more desirable to use unbiased methods to identify antigens that are implicated in immune protection.

Differential immune screening is an unbiased systems biology approach that allows for the identification of potential vaccine candidates using antibodies from semi‐immune vs susceptible subjects in endemic areas.^17^, ^18^ This approach led to the identification of UB05 (GenBank accession no. DQ235690.1, PDB PF10_0372) that was proposed as a marker of protective immunity. Another antigen, UB09, was also identified alongside UB05^17^ and later characterized.^18^ UB09 is a fragment of circumsporozoite protein‐related antigen (CRA) (GenBank accession no. XP_001347895, PDB; PF3D7_1121600,^18^ also known as exported protein‐1 (EXP‐1). In previous studies, covalently linking UB05 and UB09 produced a UB05‐09 chimera that was able to provoke interferon‐gamma production in T cells^18^ and detected enhanced antigen‐specific antibodies in semi‐immunes than malaria susceptible subjects in a significantly higher manner.^19^ Hence, it was concluded that UB05‐09 is a marker of protective immunity to malaria.

Having shown in immuno‐epidemiological studies that immune responses to UB05‐09 are associated with protective immunity against malaria, the aim of the present investigation was to seek direct proof for the concept that UB05‐09 is capable of inducing the protective immunity observed in the population studies. This was done using *Plasmodium falciparum* growth inhibition assays using rabbit antibodies to UB05‐09 and immunization‐challenge studies using the *Plasmodium yoelii* mouse model.

## 2 MATERIALS AND METHODS

### 2.1 Ethical clearance statement

The animal studies were carried out in strict accordance with the ARRIVE guidelines, and ethical clearance was obtained from the University of Buea Institutional Animal Care and Use Committee (UB‐IACUC). Ethical clearance for the collection human O+ blood for parasite culture was obtained from the Institutional Review Board of the Faculty of Health Sciences, University of Buea and the Regional Delegation of Public Health, Buea, Cameroon. Ethical clearance for blood collection and polyclonal antibody production in rabbits was approved by the ILRI Institutional Animal Care and Use Committee (ILRI‐IACUC) (ref no. 2013.05).

### 2.2 Cloning and overexpression of recombinant UB05, UB09 and UB05‐09 chimera

The study antigens recombinant UB05, UB09 and the UB05‐09 chimera were cloned and overexpressed as previously described.^17^ Briefly, the study genes were amplified from a *P. falciparum* 3D7 cDNA library and cloned into pET32a+ expression vector. The antigens were overexpressed and purified from *Escherichia coli* BL21 strain.^18^

### 2.3 Production of antigen‐specific polyclonal antibodies in rabbits

For the production of polyclonal IgG, 2 rabbits each were given UB05, UB09 or UB05‐09. Rabbits were hybrids of New Zealand × (Dutch × Grey) with the exception of 1 rabbit receiving UB05 (New Zealand × (Dutch × Chinchilla)). Rabbit selection was based on availability. Production and purification of IgG from sera specific for UB05, UB09 and UB05‐09 were as described previously.^15^ Briefly, rabbits were primed with 75 μg of recombinant antigen in Titermax adjuvant (Cat #: H4397, Sigma‐Aldrich Co. LLC.) intramuscularly. They were then boosted on days 14 and 28 by intramuscular injection with the same amount of antigen in Titermax adjuvant. The antibody titres were tested by ELISA, and when it reached 1:1000, the final boost of neat antigen was given intravenously. Five days after the neat dose, the rabbits were killed humanely and blood collected for the preparation of polyclonal antibodies. The blood was kept at room temperature for 60 minutes to allow for clotting and then centrifuged at 1500 *g* for 10 minutes. The serum supernatants were transferred into fresh tubes and heated for 60 minutes at 56°C to inactivate serum complement and make the serum suitable for neutralizing antibody assays. The inactivated serum was mixed gently and briefly and subjected to ammonium sulphate precipitation. In ammonium sulphate precipitation, 1000 g ammonium sulphate was dissolved in 1000 mL of distilled water to prepare a saturated solution. The saturated solution was added slowly to the sera samples while stirring, to a final saturation of 50%, for 30 minutes. The homogenates were spun at 3000 x g for 30 minutes and the pellets resuspended in saturated ammonium sulphate to 50% the original volume, and span again at 3000 x g/30 min. The supernatants were discarded and the pellets resuspended in PBS (in small volumes). The polyclonal antibody preparations were extensively dialysed, 3 times in PBS and the final preparations aliquoted and stored at −20°C until use. Protein concentration was determined according to the Bradford method.^20^

### 2.4 Monoclonal antibodies

Monoclonal antibodies: anti‐apical membrane antigen 1 (clone: N4‐1F6) (MRA‐481A) and anti‐erythrocyte binding antigen RII (R217 (MRA‐711A) and R218 (MRA‐712A) were obtained as a free donation from MR4/BEI Resources, NIAID, NIH, and used as positive control for growth inhibition assays as well as in combination with the rabbit antisera.

### 2.5 Parasites and animals

All laboratory strains were generously donated by the MR4/BEI Resources, NIAID, NIH, Manassas, VA. The following *P. falciparum* laboratory strains were used: 3D7 (MRA‐102, contributed by Daniel J. Carucci), FCR‐1/FVO (MRA‐909, contributed by W. Trager) and HB3 (MRA‐155, contributed by Thomas E. Wellems) for the in vitro assay. *P. yoelii* 17XL parasite was used for the in vivo studies. Two field isolates were also used for the study: GH01 from the Buea District Hospital and SC01 from the Solidarity Clinic, Buea, Cameroon. BALB/c mice were raised at the animal facility of the Biotechnology Unit of the University of Buea where they were kept in specific pathogen‐free conditions and fed on chow pellets and water ad libitum.

### 2.6 Growth inhibition assay

The ability of purified rabbit IgGs to inhibit the replication of *P. falciparum*, either singly or in various combinations, was tested by measuring parasite lactate dehydrogenase (pLDH)^21^ in late trophozoite/early schizont stage cultures as described in Methods_In_Malaria_Research 6th Edition.^22^ Briefly, after three 5% sorbitol synchronizations, enrichment of late‐stage infected erythrocytes was performed using 60% Percoll gradient centrifugation. The assay was started and stopped (≈96 hours) when most of the parasites were at the late trophozoite or schizont stage. Parasite cultures were grown at 0.1%‐0.3% parasitaemia and 1% haematocrit in a CO_2_ incubator for 2 cycles. Antisera were added at an optimized dilution of 1:10 (final concentration of anti‐UB05 total IgG = 0.63 mg/mL; anti‐UB09 total IgG = 0.67 mg/mL and anti‐UB05‐09 total IgG = 0.51 mg/mL). The monoclonal antibodies, anti‐AMA1 (final concentration of 0.1 mg/mL) and anti‐EBA‐175 RII (final concentrations; R217 at 0.133 mg/mL and R218 at 0.153 mg/mL) were used as positive controls in the inhibition assays, while negative control wells contained no antiserum or contained pre‐immune sera (final concentration of 0.7 mg/mL). The percentage of parasitaemia was determined as earlier described.^21^ There were control wells that contained either only parasitized RBCs (pRBC only) or normal RBCs. Before calculating the percentage growth inhibition, the average OD from the normal RBCs wells were subtracted from all the other OD values obtained.

### 2.7 Immunization‐challenge assay

BALB/c mice (Jackson Laboratory, CA, USA) aged 6‐8 weeks, in groups of 6, used for this study were housed at the animal facility of the Biotechnology Unit. These were introduced in our laboratory in 2006, and therefore, it has existed for 11 years of generations in Buea. They were immunized subcutaneously with 5 μg of rUB05‐09 at days 0, 21, and 42. Freund's complete adjuvant (CFA) was used in the priming dose and incomplete Freund's adjuvant (IFA) in the subsequent doses. Control groups of mice received either PBS alone or Tag fusion partner (pET32a+) alone emulsified in the same adjuvants. Twenty‐one days after the last immunization, the mice were challenged by intraperitoneal inoculation with 1 × 10^6^ *P. yoelii* 17XL infected erythrocytes obtained from a donor mouse diluted in 100 μL of PBS and monitored for blood‐stage infection. Parasitaemia was checked by microscopy from day 3 post‐infection. Treatment of a group of infected mice with Artesunate (Cat #: 1042850 USP, Sigma, Germany) was used as another positive control to suppress parasite growth in vivo.

Parasitaemia was calculated using a light microscope to count the number of infected RBCs in one thousand RBCs counted.

### 2.8 Blood and splenocytes collection for antigen‐specific IgG and interferon‐gamma production measurement

After the successful immunization assay, another immunization regime was performed as stated above for all study antigens (UB05, UB09 and UB05‐09) in parallel with PBS and Tag only as controls. Three weeks after the last boost, blood was collected for serum preparation. The sera were pooled for each group (for antigen‐specific IgG measurement by ELISA). The mice were killed, and the spleens were dissected out aseptically. Splenocytes were isolated and pooled for cell culture. For the cell culture assay, each group of splenocytes was stimulated with the antigen used for immunization for interferon‐gamma production.

### 2.9 Measurement of antigen‐specific IgG levels by ELISA

The specificity and titres of the antibodies elicited by immunization with the recombinant antigens r‐UB05‐09 chimera, r‐UB05, and r‐UB09 were determined using ELISA.^23^ Briefly, 100 μL of 5 μg/mL antigen was put in each well and incubated overnight at +4°C and then at 37°C for 2 hours with gentle shaking. Plates were blocked with 5% BSA, 0.5 %Tween 20 in PBS for 1 hour at 37°C with gentle shaking. After washing 3 times with TBST, antigen‐specific antiserum was put in each well at 1:100 dilutions and incubated for 2 hours at 37°C with gentle shaking. The plates are then washed 3 times as above and goat anti‐mouse IgG conjugated to horseradish peroxidase (Cat #: A4416‐1ML, Sigma, Germany) and put at 1:10 000 dilution and incubated for 1 hour at 37°C with gentle shaking. ABTS substrate was then added after another washing and incubated for 30 minutes in the dark and absorbance measured at 405 nm using a plate reader (Labsystems Multiskan MCC 340, Helsinki, Finland).

### 2.10 Ex vivo interferon‐gamma production

The determination of interferon‐gamma was carried out as previously described.^13^ Splenocytes were seeded ex vivo at 10 000 cells per well in a 96‐well plate in 100 μL of complete culture medium (RMPI 1640 with 12% FBS) and 100 μL of 5 μg/mL antigen. Each antigen was used to stimulate the splenocytes of the group of mice immunized with that particular antigen. After 48 hours of culture, the supernatant was collected and stored at −80°C until analysis for interferon‐gamma (IFN‐γ) concentration using mouse IFN‐γ ELISA kit from Sigma according to manufacturer's instructions. Splenocytes were also isolated from the group of rUB05‐09‐immunized mice 4 months after they survived a lethal challenge infection with *P. yoelii* 17XL.

### 2.11 Statistical analyses

All statistical tests were carried out using SPSS software version 17.0 (SPSS Inc. Chicago, IL, USA). The nonparametric Kruskal‐Wallis test was used to determine the significance of differences in parasitaemia due to immunization with the study antigens. ANOVA was used to assess the effect of the various IgGs on parasite growth and the effect of immunization on antibody and IFN‐γ production. *P* values equal to or below .05 were considered significant.

## 3 RESULTS

### 3.1 Antibodies to the chimera UB05‐09 perform better in inhibiting parasite growth than antibodies to the respective constituent components

**Figure 1:**
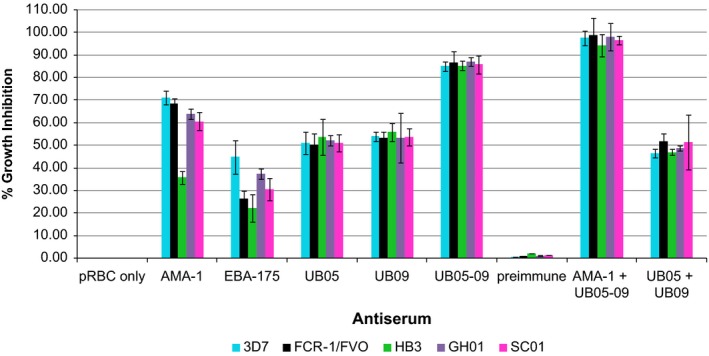
Growth inhibition assay. Rabbit antisera against rUB05, rUB09 and rUB05‐09 chimera were used in vitro to test for their ability to inhibit parasite growth. This was done using *Plasmodium falciparum* laboratory strains; 3D7, FCR‐1/FV0 and HB3 and 2 field isolates; GH01 and SC01. Antisera were combined in various combinations and tested for their ability to inhibit parasite growth in vitro. They were tested at 1:10 dilution. Error bars indicate standard deviation. The experiment was carried out twice in triplicates

The ability of the antigen‐specific antisera to inhibit parasite growth in vitro was tested using the growth inhibition assay (GIA) as described in Materials and Methods. Rabbit anti‐UB05‐09 antiserum was able to inhibit parasite growth in vitro in a way that was significantly higher than the performance of anti‐UB05 (*P *=* *.0001) or anti‐UB09 antiserum (*P *=* *.0001) (Figure 1). There was no difference in the ability of both anti‐UB05 and anti‐UB09 antisera to inhibit in vitro parasite growth. This trend was observed for all the parasite strains tested (Figure 1). When the positive controls (anti‐AMA1 and anti‐EBA175 antibodies) were tested, the growth of *P. falciparum* HB3 strain was not inhibited in contrast to the other parasite strains studied whose growths were significantly inhibited. Anti‐UB05‐09 antiserum inhibited parasite growth more than anti‐AMA1 (*P *=* *.003) even though not significantly, but significantly higher than anti‐EBA175 (*P *=* *.0001) antibodies. No significant inhibition was observed in the wells that contained the pre‐immune sera.

To find out if this enhanced performance was not due to the fact that the antiserum was raised against the covalently linked UB05‐09 chimera, experiments were designed to test whether the same levels of inhibition could be attained by simply mixing anti‐UB05 and anti‐UB09 antisera that were raised separately. It was observed that simply mixing the 2 antisera in 1:10 dilutions did not inhibit parasite growth to the level observed with anti‐UB05‐09 antiserum. This was observed for all parasite strains tested (Figure 1).

### 3.2 A mixture of anti‐AMA1 and anti‐UB05‐09 antisera completely inhibited in vitro *P. falciparum* growth

In a bid to enhance the parasite growth inhibition effect of anti‐UB05‐09 antiserum, it was tested in combination with other antibodies raised against some well‐studied malaria vaccine candidates. An additive effect was observed when anti‐AMA1 and anti‐UB05‐09 antibodies were combined and tested at a 1:10 dilution. This led to complete inhibition of parasite growth in vitro. This complete inhibition was obviously greater than the inhibition observed with either anti‐AMA1 or anti‐UB05‐09 antibodies alone (*P *=* *.0001) (Figure 1). No enhanced inhibition was observed when anti‐UB05‐09 and anti‐EBA175 antibodies were combined and tested together (Figure 1).

### 3.3 Immunization of mice with rUB05‐09 completely protected the mice against lethal *P. yoelii* 17XL challenge infections

**Figure 2:**
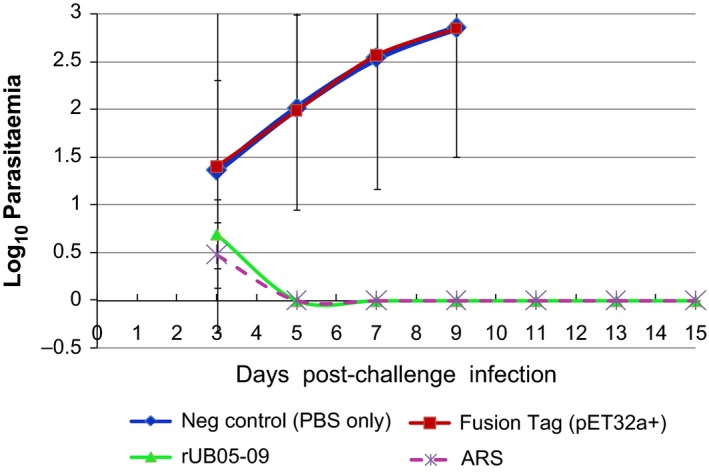
Immunization assay using recombinant UB05‐09 and Tag fusion partner (pET32a+). BALB/c mice (groups of 6) were immunized with 5 μg of either PBS, Tag fusion protein or recombinant UB05‐09 09 and challenged with a lethal *Plasmodium yoelii* 17XL infection. Freund's complete adjuvant was used for priming and Freund's incomplete adjuvant for the booster doses. Mice immunized with rUB05‐09 were completely protected from murine malaria (*P *=* *.018). Artesunate treatment of mice was used as a positive control to prevent parasite growth in vivo. The experiment was carried out twice and the average presented

After observing an additive effect on the inhibition of in vitro parasite growth, immunization of mice with rUB05‐09 was carried out to test whether it can confer protection to the immunized mice against a lethal *P. yoelii* 17XL challenge infection. Each group of mice contained 6 animals. The group of mice immunized with rUB05‐09 developed a low transient parasitaemia by day 3 which was cleared by day 5, indicating that the mice successfully cleared parasitaemia (*P *=* *.018) (Figure 2). By contrast, the control mice in the group that received PBS or Tag protein emulsified in CFA/IFA all developed parasitaemia that reached 70% by day 9 and the mice had to be killed humanely to prevent suffering. The positive control group that received artesunate developed low transient parasitaemia by day 3, which was rapidly cleared (Figure 2). The experiment was repeated once.

### 3.4 Protection of immunized mice against lethal parasite infection involves specific antibody production

**Figure 3:**
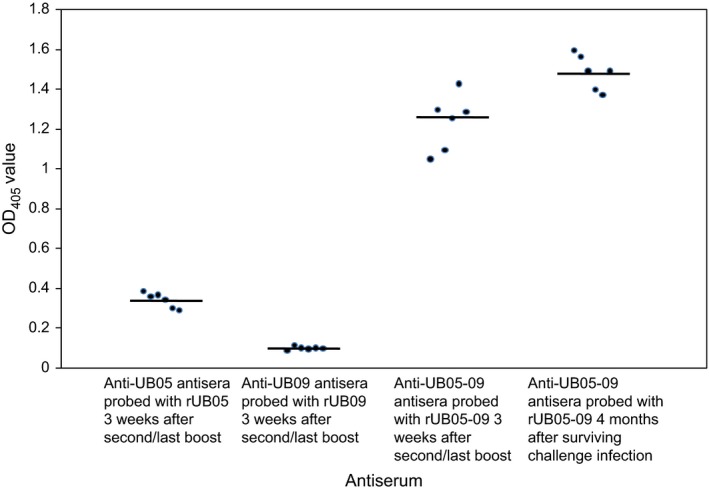
ELISA to detect antigen‐specific antibody. Sera were collected from mice either 3 wk after the second/last boost or 4 mo after surviving a lethal challenge malaria infection and probed with the recombinant antigen used for immunization. rUB05‐09 synergistically enhanced antigen‐specific antibody levels (*P *=* *.0001). Sera from pre‐immunized mice and mice immunized with either the fusion partner (Tag only) or PBS emulsified in CFA/IFA were used as controls and showed little or no antibody levels and used as background signal. This was done twice in triplicates

After noticing that immunization with rUB05‐09 completely protected the mice against lethal challenge infection, the immunization regime was repeated to identify the arm(s) of the immune system that could be involved. It was observed that antigen‐specific antibody levels induced by rUB05‐09 were greater than that of rUB05 as measured by ELISA and far greater that of those induced by rUB09 (*P *=* *.0001), suggesting a synergistic effect (Figure 3).

When sera collected from mice that had survived the challenge infection for 4 months were analysed for Immunoglobulin‐G levels, it was observed that the antigen‐specific antibody for rUB05‐09 was significantly higher than that for rUB05 and far higher than that for rUB09 (*P *=* *.0001) (Figure 3). It was also observed that 4 months after surviving a lethal *P. yoelii* 17XL challenge, the anti‐UB05‐09 antibody level was higher than in the sera obtained from mice 3 weeks post‐second/last boost, and this was statistically significant (*P *=* *.004) (Figure 3). Sera from mice immunized with either the Fusion partner (Tag only) or PBS alone showed no detectable antibodies. Furthermore, pre‐immune sera showed only background signal (data not shown).

### 3.5 Anti‐UB5‐09 antiserum recognized mostly rUB05

**Figure 4:**
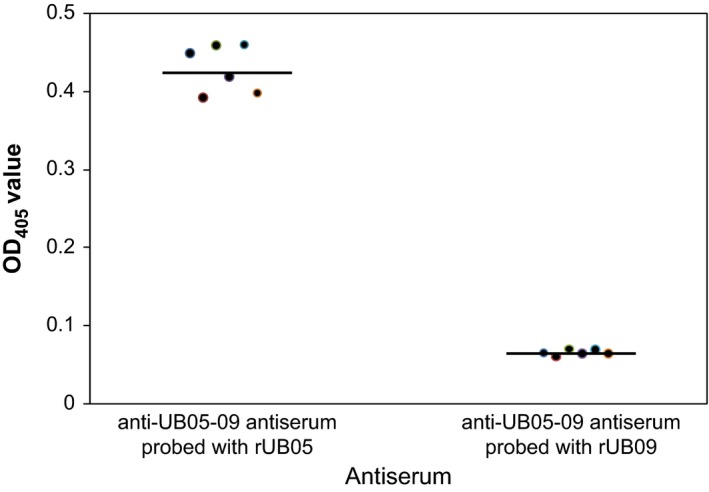
Anti‐UB05‐09 antiserum probed with rUB05 and rUB09. Antiserum from mice immunized with rUB05‐09 was collected 3 wk after the second/last boost and probed with either rUB05 or rUB09 using ELISA. The antiserum contained mostly rUB05 antibodies compared to rUB09 antibodies (*P *=* *.0001). This was done twice in triplicates

To find out if anti‐UB05‐09 antiserum can recognize both UB05 and UB09, the antiserum was probed with rUB05 and rUB09 by ELISA and it was observed that the anti‐UB05‐09 antisera from immunized mice recognized mainly rUB05 and very little of rUB09 (*P *=* *.0001) (Figure 4). These results are consistent with Western blot data that showed anti‐UB05‐09 antiserum raised in rabbits preferentially binds rUB05 over rUB09 (data not shown).

### 3.6 Interferon‐gamma production is stimulated in mice vaccinated with UB05‐09 against *P. yoelii* infection, but not in control mice

**Figure 5:**
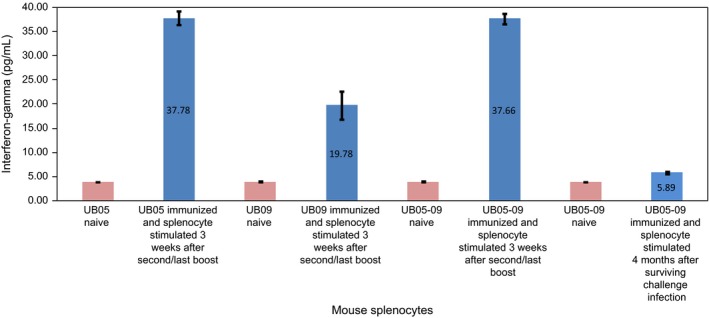
Mouse splenocytes stimulated ex vivo for interferon‐gamma production. Splenocytes were aseptically collected from immunized mice either 3 wk after the second/last boost or 4 mo after surviving a lethal challenge *Plasmodium yoelii* 17XL infection. These were stimulated ex vivo with the same antigen used for the immunization regime. Interferon‐gamma production was significantly higher in immunized mice compared to naïve mice (*P *=* *.0001). The experiment was carried out twice in triplicates

To find out if IFN‐γ production occured was during vaccination with rUB05‐09, splenocytes were aseptically collected from mice immunized with rUB05‐09, 3 weeks after the second/last boost and also from immunized mice that were 4 months into surviving a lethal *P. yoelii* 17XL challenge infection. After stimulating the splenocytes ex vivo with the various study antigens, it was observed that the splenocytes from all the immunized mice produced significantly higher levels of IFN‐γ than splenocytes from naïve mice (*P *=* *.0001) (Figure 5) which served as controls. However, the level of IFN‐γ produced by splenocytes collected from mice that survived the challenge infection 4 months earlier was significantly lower compared to those collected 3 weeks after second/last boost (*P *=* *.0001) (Figure 5).

## 4 DISCUSSION

The present investigation shows clearly for the first time that the *P. falciparum* chimeric antigen construct UB05‐09 can induce immune protection against *P. yoelii* challenge infection in mice.

We have previously shown in immuno‐epidemiological studies that immune responses to chimeric *P. falciparum* UB05‐09 antigen are associated with protective immunity against malaria and the immune responses observed to UB05 and UB09 could be enhanced by covalently linking the 2 proteins to produce a more immunogenic antigen. In the present proof‐of‐concept study, we provide experimental evidence to buttress the status of UB05‐09 as a malaria vaccine candidate as follows:

Antibody against the chimeric UB05‐09 antigen showed a strong parasite growth inhibitory activity using a two‐cycle growth inhibition assay. This activity was significantly greater than that observed with either UB05 or UB09 alone. Furthermore, simply combining anti‐UB05 and anti‐UB09 antisera did not exhibit an additive effect. This indicates that producing a potent parasite growth inhibitory antibody required a preformed UB05‐UB09 complex. It has been predicted that CRA interacts with UB05 in vivo^24^, ^25^ and this interaction could lead to the creation of additional epitopes, or enhancement of the immunogenicity of existing epitopes. Conceivably, the covalent linkage of UB05‐09 chimera could have resulted in new potent epitopes found neither on UB05 nor UB09, but this needs to be verified by additional structural studies.

The growth inhibitory activity of anti‐UB05‐09 antisera was greater than that of the well‐studied vaccine candidate, the apical membrane antigen (AMA1), even though this was not significant. However, combining anti‐UB05‐09 and anti‐AMA1 antibodies completely inhibited parasite growth in vitro. The ability of combined anti‐UB05‐09 and anti‐AMA1 antibodies to completely inhibit in vitro parasite growth indicates that their action is synergistic and both may be acting through 2 different pathways that are vital to the parasite. Hence, the efficacy of a subunit vaccine could be enhanced by including multiple components serving different crucial functions in the parasite.

After demonstrating the potent in vitro activity of anti‐UB05‐09 antiserum, we decided to immunize mice with chimeric *P. falciparum* UB05‐09 antigen and challenge them with a lethal *P. yoelii* 17XL infection. The group of mice immunized with rUB05‐09 completely survived the lethal challenge infection indicating that the antigen was able to create enough immune memory to completely protect the immunized mice. It is unlikely that these results could be due to endotoxin contamination because in the control experiments, the tag fusion partner which was overexpressed and purified under the same conditions as the test antigens, the background was generally low all through. Furthermore, the fusion partner gave little or no background signals. Hence, values obtained for test antigens were specific. The protein purity and homogeneity was checked by SDS‐PAGE to be approaching homogeneity as earlier published.^18^

In an attempt to elucidate the mechanisms involved in the immune protection induced by UB05‐09 and its constituent antigens, we measured antigen‐specific antibodies in the sera from immunized mice and found that there was a synergistic effect by the amount of antibodies induced by UB05‐09 compared to UB05 or UB09 alone. Sera that were collected from mice 4 months after they had survived a challenge infection contained anti‐UB05‐09 antibodies amounts higher than in the sera collected from mice 3 weeks after the second/last boost with rUB05‐09 antigen even though this increment was statistically significant. It appears that the humoral response induced by rUB05‐09 was long‐lasting in mice. Additional experiments are needed to verify this point.

There appeared to be a delayed development of a cellular immune response to *Plasmodium* infection, but it is not known whether this belated development of cellular immune response occurs after the acquisition of protective immunity or acquiring protection is associated with the development of T‐cell response. A study by D'Ombrain et al^26^ showed that semi‐immune older children whose T cells produced the highest amount of IFN‐γ in vitro were far less likely to develop a high‐density parasite infection for up to another 6 months. UB05‐09 was able to induce IFN‐g production, but its role in the observed protection remains to be determined.

In another approach to develop an effective malaria vaccine, it will be advantageous to identify antigens that can provoke a protective immune response against different parasite strains and/or species.^27^ This is particularly useful as there are reports of several human plasmodia species coexisting in malaria‐endemic regions. The treatment of one species could have severe clinical consequences emanating from a rebound effect or increase in the prevalence of a parasite previously kept under control.^28^ The observation that *P. falciparum* antigen construct UB05‐09 could induce protective immune responses in mice against *P. yoelii* infection suggests the capacity of this antigen to exert cross‐species regulatory effects. This is compatible with the fact that the antigen UB05 from *P. falciparum* shares 70% identity and 85% homology with its orthologue in *P. yoelii* while CRA (of which UB09 is a fragment) in *P. falciparum* has 56% identity and 70% homology with its orthologue in *P. yoelii* (PY17X_0522000).^29^ It also indicates that the chimeric UB05‐09 antigen possesses highly conserved epitopes that could be effective in vaccinating against a broad spectrum of plasmodium parasites. These data are consistent with the possibility of cross‐vaccination which is a well‐known phenomenon as the case of BCG vaccine.^30^ Furthermore, as CRA, a blood‐stage *P. falciparum* antigen, is expressed in the liver stage of the plasmodium parasites,^31^ it is conceivable that immune responses provoked by UB05‐09 could target the pre‐erythrocytic stages of the parasite as well, but this remains to be proven.

Although anti‐UB09 IgG was able to partially inhibit in vitro parasite growth and CRA (Exp‐1) has been predicted to interact with UB05 during blood stage of the parasite life cycle, it is not still clear how cytoplasmic antigens are able to induce protective immune responses. However, it has been proven that cytoplasmic and intracellular antigens could be developed as candidate vaccine antigens.^32^ It has also been suggested that an antigen with multiple epitopes may contain both protection‐inducing and inflammatory‐inducing epitopes^13^; even though UB09 is only a fragment of CRA, it may well contain protection‐inducing epitope(s).

It has been shown that protective immunity provoked by whole organism immunization indicates the accumulation of small responses to a large number of antigens; likewise, a single antigen with one or more epitopes could be enough to illicit a protective immune response when presented to the immune system in an appropriate manner.^33^ UB05‐09 is composed of 2 antigen fragments with multiple B‐ and T‐cell epitopes as shown in previous bioinformatics analyses.^18^, ^19^ It is therefore capable of stimulating both arms of the immune system to provide protection against *P. yoelii* infection. Whether or not UB05‐09 will be effective in vaccinating against *P. falciparum* malaria in humans remains to be seen. However, the trans‐species efficacy of UB05‐09 as demonstrated herein strongly suggests that this is possible.

The growth inhibition assay alone has been considered sufficient functional assays for blood‐stage vaccine development^7^ and has been used to identify malaria vaccine candidates without resorting to other assays. Similarly, immunization/challenge infection studies alone have also used to identify malaria vaccine candidates.^8^, ^34^ Herein, we present both types of experiments which strengthen the probability that UB05‐09 could be a malaria vaccine candidate.

In conclusion, the serologic, cytokine and in vivo studies presented herein strongly show that chimeric *P. falciparum* UB05‐09 antigen is a malaria vaccine candidate worthy of further study.

## CONFLICTS OF INTEREST

None.

## AUTHOR'S CONTRIBUTION

JND conceived the project. JND, VPKT and GSD performed the experiments. JND, VPKT and GSD performed the experiments. JND, VPKT and SMG analysed the data. JND, VPKT, GSD and SMG drafted the manuscript or revised it critically for important intellectual content. JND and VPKT agree to be accountable for all aspects of the work. All authors read and approved the final manuscript.
